# “It´s incredible how much I´ve had to fight.” Negotiating medical uncertainty in clinical encounters

**DOI:** 10.1080/17482631.2017.1392219

**Published:** 2017-10-24

**Authors:** Olaug S. Lian, Catherine Robson

**Affiliations:** ^a^ Department of Community Medicine, Faculty of health sciences, University of Tromsø – The Arctic University of Norway, Tromsø, Norway

**Keywords:** Medical uncertainty, myalgic encephalomyelitis, medically unexplained physical symptoms, doctor-patient interactions, patient-centered care, patient perspectives

## Abstract

**Purpose:** Clinical encounters related to medically unexplained physical symptoms (MUPS) are associated with high levels of conflict between patients and doctors. Collaborative difficulties are fused by the medical uncertainty that dominates these consultations. The main aim of this study is to explore the interactional dynamics of clinical encounters riddled by medical uncertainty, as experienced by people living with long-term medically unexplained fatigue in Norway. **Method:** A qualitative thematic analysis of written texts from 256 study participants. **Results:** We found that patients experience being met with disbelief, inappropriate psychological explanations, marginalisation of experiences, disrespectful treatment, lack of physical examination and damaging health advice. The main source of their discontent is not the lack of biomedical knowledge, but doctors who fail to communicate acknowledgement of patients’ experiences, knowledge and autonomy. War metaphors are emblematic of how participants describe their medical encounters. The overarching storyline depicts experiences of being caught in a power struggle with doctors and health systems, fused by a lack of common conceptual ground. **Conclusion:** When physical symptoms cannot be detected, explained and managed by biomedical knowledge and technology, good doctor-patient partnerships are crucial. Without clearly acknowledging patients’ perspectives and capabilities in clinical practice, such partnerships cannot be achieved.

## Introduction

Medical uncertainty—understood as a state of doubt and ambiguity about the aetiology, diagnosis, treatment, and/or prognosis of illness—is an inherent quality of all biomedical knowledge and clinical practice, to various degrees (Han, Klein, & Arora, 2011). Somatic symptoms without discernible organic pathology, or so-called medically unexplained physical symptoms (MUPS), score high on all accounts.

In clinical consultations epitomized by great uncertainty, therapeutic relationships between patient and doctor become paramount (Kornelsen, Atkins, Brownell, & Woollard, 2016). Such partnerships, however, are difficult to achieve: medical encounters related to MUPS are associated with conflicts between patients and doctors who struggle to cooperate with each other (Dowrick et al., 2008; Lian & Nettleton, 2015; Libert et al., 2016). For doctors who are trained to work on the basis of evidence-based biomedical knowledge and technological proof, somatic symptoms unsubstantiated by observable biomarkers to verify organic disease are challenging. When their ability to identify, explain and treat patients’ ailments is constrained by an uncertain biomedical foundation, they often feel powerless, inadequate, dissatisfied, frustrated and anxious (Åsbring & Närvänen, 2003; Chew-Graham, Cahill, Dowrick, Wearden, & Peters, 2008; Chew-Graham, Dowrick, Wearden, Richardson, & Peters, 2010; Howman, Walters, Rosenthal, Ajjawi, & Buszewicz, 2016; Libert et al., 2016; Murray, Toussaint, Althaus, & Löwe, 2016). For patients, experiences of uncertainty can increase psychological distress, intensify sensitivity to pain (Rosendal et al., 2013; Taylor, Marshall, Mann, & Goldberg, 2012; Weiland et al., 2012; Wright, Afari, & Zautra, 2009), and result in poorer health (Neville, 2003), reduced quality of life and diminished confidence (Ogden et al., 2002).

Interaction and communication between patient and doctor in clinical encounters are integral to how health services work (King & Hoppe, 2013). Strong and collaborative doctor-patient partnerships can mitigate the challenges brought by the lack of clarity, and thereby reduce detrimental effects of medical uncertainty (Kornelsen et al., 2016). Unveiling factors that support and hinder doctor-patient partnerships is therefore important.

In this article, we explore doctor-patient partnerships in the territory of medical uncertainty. Most importantly, we seek to explore the dilemma of institutionalized expertise versus individual rights of self-determination by studying the power-related interactional dynamics that arise in consultations characterized by medical uncertainty. Our data are limited to healthcare experiences of people with medically unexplained long-term fatigue, often labelled myalgic encephalomyelitis (ME), and our main research questions are: as patients, how do they experience the negotiation of power in medical encounters fused by medical uncertainty, and what is the main outcome of these negotiations?

ME is an illness surrounded by many medical uncertainties. The condition is currently described as a post-exertional fatigue (duration at least six months) that does not disappear after resting (Carruthers et al., 2011). The fatigue (a severe form of tiredness) is usually accompanied by malaise, dysregulation of body temperature, bowel problems, sleep disturbances, and concentration difficulties (Carruthers et al., 2011). Interchangeable labels used to describe the condition include chronic fatigue syndrome (CFS) and post-viral fatigue syndrome (PVFS). The World Health Organisation classifies ME as a neuro-immunological condition (WHO ICD, 2016), but many doctors consider it a somatic symptom underpinned by psychological causes, predominantly stress (Wyller, Eriksen, & Malterud, 2009). Despite the promising identification of biological abnormalities in recent years (Frémont, Coomans, Massart, & De Meirleir, 2013; Giloteaux et al., 2016), there are, as yet, no observable biomarkers to verify organic disease. Of those who receive the diagnosis, around 75–85% are women (Capelli et al., 2010; Faro et al., 2016).

Our study is based on data from a national survey of patient experiences of publicly funded healthcare services among members of the Norwegian ME Association (Hansen & Lian, 2016a, 2016b; Lian & Hansen, 2016) where we asked study participants to elaborate their experiences in free-text comments. Based on an inductive, thematic and qualitative study of these texts we explore doctors’ handling of uncertainty in medical encounters—as patients perceive it. By eliciting and exploring healthcare experiences among patients with medically unexplained long-term fatigue we hope to increase our knowledge about how patients perceive the power dynamics of medical consultations, and the interactional challenges both parties encounter when facing medical uncertainty. After presenting our data, structured according to six themes drawn inductively from the data, we situate our participants’ experiences in their cultural context, and discuss the handling of medical uncertainty in doctor-patient partnerships in relation to previous research.

## Methods

Data were gathered from an email survey conducted in April-May 2013. Statistical data from sub-sets of female participants have previously been published (Hansen & Lian, 2016a, 2016b; Lian & Hansen, 2016).

### Participants

Invitations to participate were distributed to 811 members of the Norwegian ME Association with known email addresses (about 40% of all members). Members were asked to refrain from participating if they were below the age of 16 years or did not suffer from the condition themselves (health professionals, parents, others). Four hundred and eighty-eight people (60% of those approached) submitted a return. We do not know how many people approached were not eligible to participate; consequently, an exact response rate cannot be calculated. Of the 488 respondents, we excluded 22 who did not give information about their age, gender or diagnosis. Of the remaining 466, 256 participants (55%) responded to the following open-ended question in the survey: “Is there something that you want to tell us that you have not already done so far in this survey?” These 256 respondents are the participants of this study.

### Data-analysis

All free-text comments were gathered in one document, amounting to 26,500 words (around 100 words per respondent). After removing all identifying information, the texts were translated from Norwegian to English by a professional (native English) translator. Translated texts were checked for consistency and accuracy by the first (native Norwegian) author.

Texts were analysed in collaboration between both authors using an inductive qualitative thematic approach (Braun & Clarke, 2006) inspired by a grounded theory strategy (Charmaz, 2014; Glaser & Strauss, 1967) and a constant comparative method (Glaser & Strauss, 1967). We developed our research questions after identifying patterns observed while exploring all 256 texts, and themes for the analysis were drawn inductively from the data. In stage 1 of the analysis we identified as many themes as possible that emerged across the dataset (no themes were defined in advance). In stage 2, we classified textual meaning units (the smallest text units that we coded) based on our list of themes consistently for all texts. During this phase we continuously discussed and revised our interpretations and classifications, eventually settling on a list of six themes to concentrate on (numbered 1–6 in Results section). Using our data-driven codes, we then classified data extracts belonging to these six themes, and analysed the data systematically, first individually and then both of us together. While searching for both manifest and latent meaning of the texts, the interrogative pronouns “what”, “how”, “who” and “why” were key words. Decondensation of key concepts used in the texts was a vital part of this process; for instance, what do they mean by “being understood”, and how does this relate to “being believed”? Designation of texts involved balancing different considerations. Splitting long quotes, for instance, reduces the challenge of “multi-thematic” extracts, but also increases the risk of losing context. To ensure trustworthiness and theoretical saturation, we formulated and reformulated competing interpretations several times through an exploratory and case-led approach, and we rechecked our reading several times.

To compare the sociodemographic and health characteristics of participants who provided free-text comments with those who did not, we conducted a statistical analysis using descriptive methods and non-parametric tests of significance and correlation (Tables I and II).

**Table I.** Responders versus non-responders—sociodemographic and health characteristics.

Variable	Respondents who wrote free-text comments (N=256) (Number/percent)	Respondents who did *not* write free-text comments (N=210) (Number/percent)	P-value
**Gender**	**256 (100 %)**	**210 (100 %)**	0.55*
- Women	230 (90 %)	185	
- Men	26 (10 %)	25	
**Age (years)**	**256 (100 %)**	**210 (100 %)**	0.12^+^
- Range	16 to 72	17 to 73	
- Median	47	45	
**Education**	**256 (100 %)**	**194 (92 %)**	0.19^
- Primary school	8 %	8 %	
- High school	29 %	35 %	
- Undergraduate	38 %	41 %	
- Post-graduate	25 %	16 %	
- Median value	Undergraduate	Undergraduate	
**General health**	**256 (100 %)**	**194 (92 %)**	0.25^+^
- Very good	1 %	1 %	
- Good	9 %	10 %	
- Neither good/poor	19 %	23 %	
- Poor	52 %	52 %	
- Very poor	19 %	14 %	
- Median value	Poor	Poor	
**Severity of ME**	**254 (99 %)**	**194 (92 %)**	0.23^+^
- Mild	19 %	23 %	
- Moderate	70 %	68 %	
- Severe	10 %	8 %	
- Very severe	1 %	1 %	
- Median value	Moderate	Moderate	
**Age of illness onset (years)**	**245 (96 %)**	**205 (98 %)**	0.19^+^
- Range	10 to 61	11 to 62	
- Median	35	33	
**Diagnosis (multiple responses)**	**256 (100 %)**	**210 (100 %)**	
- ME	77 %	78 %	0.32*
- CFS	8 %	6 %	0.61*
- PVFS	15 %	16 %	0.72*
**Changed GP last 12 months**	**255 (99 %)**	**200 (95 %)**	0.32*
- Yes	25 %	21 %	
- No	75 %	79 %	
**Time with current GP if not changed last 12 months (years)**	**187 (98 % of those eligible)**	**155 (98 % of those eligible)**	0.13^+^
- Range	1 to 35	1 to 35	
- Median	6.5	10.5	
**Number of health visits last 12 months for ME**	**237 (93 %)**	**180 (86 %)**	0.29^+^
- Range	0 to 50	0 to 25	
- Median	4	4	
**Private health utilization last 12 months for ME**	**231 (90 %)**	**181 (86 %)**	0.54*
- Yes	38 %	35 %	
- No	62 %	65 %	

* Fisher´s Exact Test (2-tailed)

^ Fisher´s Exact Test Freeman-Halton Extension (2-tailed)

^+^ Mann-Whitney U Test (2-tailed)

**Table II.** Responders versus non-responders: GP satisfaction

VARIABLE	Respondents who wrote free-text comments (N=256) (Number/percent)	Respondents who did *not* wrote free-text comments (N=210) (Number/percent)	P-value
**Degree of satisfaction with *initial* help from GPs (for ME)**	**255 (99 %)**	**204 (97 %)**	0.01^+^
- To a large extent	18 %	23 %	
- To some extent	22 %	33 %	
- To a little extent	32 %	25 %	
- Not at all	29 %	19 %	
Degree of satisfaction with help from *current* GP (for ME) *	**240 (94 %)**	**185 (88 %)**	0.07^+^
- To a large extent	20 %	25 %	
- To some extent	54 %	57 %	
- To a little extent	23 %	15 %	
- Not at all	3 %	3 %	
**Test of equal distribution: satisfaction with initial versus current help**	**240 (94 %)**	**185 (88 %)**	>0.001^§^

^+^ Mann-Whitney U Test (2-tailed)

^§^ Wilcoxon Signed-Rank Test (2-tailed)

* Proxy measure; median result of seven combined measures that used the same (4 point) Likert scale: In relation to ME/CFS/PVFS, in the last 12 months has your current GP 1) taken you and your issues seriously, 2) respected what is important to you as a patient, 3) shown a mutual understanding of your illness, 4) listened closely to what you have to say, 5) sought approval for decision making, 6) provided advice for problems, and 7) provided understandable information?

### Ethics

Ethics approval was granted by the Norwegian Data Protection Official (id. 31,784). The invitation to participate contained information about the purpose of the study, and measures taken to ensure participants’ anonymity. We also informed participants that they could withdraw from the study at any time should they wish to do so. Informed consent, including consent for publication, was obtained from all participants.

## Results

Study participants were mainly women (90%) and highly educated (63% at university level). Most of them reported having an ME diagnosis (75%) and a moderate degree of fatigue (70%). The sociodemographic and health characteristics of those who provided free-text comments were not significantly different from those who did not (Table I). Compared to those who did not provide free-text comments, free-text respondents reported significantly lower levels of satisfaction with general practitioners (GPs) shortly after illness onset (Table II). However, a statistically significant increase in the level of GP satisfaction over time was found for both groups (Table II).

In the following, we present and summarize text extracts related to six main themes. Participant IDs refer to the degree of participant’s fatigue (MI = mild, MO = moderate, SE = severe and VS = very severe) and unique identifying number. To keep the voice of participants to the forefront we present the data with minimum interference, before turning to the discussion.

### Theme 1: lack of medical knowledge

Our participants experience a lack of medical knowledge about their condition, particularly within primary care:

“The primary health service knows little about fatigue-related conditions (SE-7302).”

“At times I have been met with great scepticism about my illness and zero knowledge about it (MO-7501).”

These statements are often formulated as some kind of critique, for instance by the use of words like “rude” and “prejudice”:

“It is upsetting how little knowledge the health services have about ME and how rude they are; they have no knowledge of the illness and don’t listen to the patient at all (MI-7562).”

“… the extreme prejudice and lack of knowledge one encounters with ME (MO-7107).”

In the absence of certainty through medical knowledge, they call for doctors who listen to patients, and respect and acknowledge patients’ experiential knowledge:

“It is I who know my body and what happens when I am overstrained, socially, mentally and physically […] I can’t expect my GP to have this knowledge, but I can expect my GP to listen to what I have to say and respect me as a person (SE-7302).”

Participants also reflect on doctors’ helplessness that stems from this lack of knowledge:

“… the GP […] felt helpless and didn’t know what to do (MO-7620).”

“… my GP […] examined me thoroughly and maintained that she couldn’t help me because ‘there are so many like you and we don’t know what to do with you’ (MI-7337).”

### Theme 2: not being understood

Participants seem to distinguish between knowledge and understanding. They relate knowledge to biomedical facts, whereas understanding is more about whether or not doctors acknowledge patient experiences. They describe a lack of understanding in general terms, as existing among the medical profession (and sometimes broader society) as a whole, or they refer to specific doctors:

“… met with zero help and zero understanding (MO-7571).”

“I encounter little understanding and a number of preconceptions (MI-7425).”

“The loneliness and helplessness one experiences as a sufferer is reinforced by the feeling of not being understood (MO-7163)”

“… the meeting with neurologists (specialists) was characterized by a lack of understanding (MO-7223).”

“… she [GP] has little understanding of my ME (MO-7074).”

Lack of understanding is sometimes accompanied by disrespectful behaviour:

“The first GP was so dreadful and unwelcoming, actually ridiculed ME (MO-7372).”

“I was downright shouted at [by the neurologist] and referred to a psychiatrist (MO-7223).”

“One literally yelled at me and physically threw me out of his office after throwing something at me from his desk (SE-7066).”

### Theme 3: being disbelieved

Participants also distinguish between “being understood” and “being believed”. They report widespread disbelief among health professionals both of the existence of ME, and of patient experiences. When described in generalized terms, they refer to doctors as a group, the health system, or medicine as a whole:

“Some doctors didn’t ‘believe’ in the illness (SE-7066).”

“I fell ill in 1996 and at that time ME was an illness that was taken seriously, now it’s all seen as nonsense (MO-7428).”

“they didn’t believe my situation (MO-7199).”

However, they more frequently recount specific encounters with individual health professionals, often reporting what the health professional had told them:

“I had dreadful encounters with previous GPs, was disbelieved and wrongly treated and absolutely not listened to (MO-7143).”

“… a psychiatrist (cognitive therapy) […] said straight out that ME didn’t exist. I became incredibly unwell during that period (MO-7469).”

“The GP didn’t believe me […] My GP said that ME didn’t exist (MO-7152).”

“His own opinion was that ME didn’t exist (MO-7226).”

“My GP threw a fit from the first day I asked him whether I might have ME. He had never heard of it, and when I said its full name, he shouted that I couldn’t have that in any case, because it meant an infection of the cerebrospinal fluid […] He yelled at me that this was a fake disease (SE-7472).”

Doctors seem to transfer their scepticism towards the disease as such to patients’ illness experiences, and so these experiences become disbelieved and discredited:

“It was absolutely shocking for me not to be believed when I was so poorly (ME-7139).”

“I wish that ME was respected as an illness by the healthcare system so that we would no longer be met with distrust (MO-7515).”

“I had to stop going to see the first one because she absolutely refused to believe that I was ill (MI-7139).”

“… the doctor I had after that was a man, and didn’t take me so seriously (MO-7440).”

The word “hypochondriac” features in several descriptions:

“For all these years my GP has treated me like a hypochondriac (MO-7665).”

“The [nationality] doctor said that I was far too young to be ill, […] concluded that I was a hypochondriac, diagnosis neurasthenia, and in a letter to my GP he wrote that under no circumstances must I be referred back to him, but should rather be referred to a psychologist (MO-7462).”

Participants also describe how doctors distrust their expressed experiences of suggested treatments:

“She said that she had never heard of ‘anyone getting ill from going for a walk’ (MO-7645).”

“She [GP] suggested some physical activity because she ‘doesn’t believe that one can get well by resting—not from ME either’, as she said […] she wouldn’t understand when I told her how this activity thing affects me […] The result comes the following day, or for days after: I get terrible pains in my body and debilitating fatigue, and have to lie in bed for two to three days (MO-7645).”

### Theme 4: lack of congruence on explanations

Participants complain about doctors who reduce their ailment to a mental condition, and therefore disregard its physicality. Participants object to this interpretation because they experience the illness as “fairly concrete in physical terms (MO-7163)”:

“The fact that the medical profession won’t believe that a person is physically ill is awful!!! (SE-7228).”

“They interpret it as mental, which represents a direct risk for ME sufferers (SE-7781).”

“Some assume that it’s a mental illness (MI-7425).”

More frequently, participants draw on personal experiences to describe how psychological theories serve—as they perceive it—to dismiss or diminish their experiences of physical symptoms, and sometimes even question their personal character:

“I have had doctors at the hospital who laugh at me. They ask if I am one of those stressed housewife types before I have even opened my mouth. Or they explain it as mental (MO-7202).”

“I feel that my new GP thinks I am lazy and anxious (MO-7645).”

“I was treated extremely badly and disdainfully. The next one examined me willingly, but laughed at both my ME diagnoses and fibromyalgia diagnoses. Called it a bunch of crazy people (SE-7554).”

“I wasn’t taken seriously by my GP [when I fell ill…] brushed aside by my GP who told me it was attributable to unhappiness (so the cause was mental) (MO-7803).”

Participants also recount how a lack of assumed “objective” signs of illness in the form of organic pathological findings or physical appearance, are used by doctors to diminish their physical symptoms:

“I was told by a specialist I was referred to that now I had been examined ‘from head to arse’, so now I finally had to understand that there was nothing [physically] wrong with me (MO-7090).”

“… the doctor did not inform me that he assumed it was mental […] In the end, I confronted my GP with ‘you’re not listening to what I’m telling you’, and he replied, ‘no, I see how you appear’ […] and you don’t appear to be ill (MO-7362).”

Participants reject the Cartesian dualistic approach to psyche and soma that dominates the health system, including the tendency to treat symptoms in isolation, not holistically:

“I experience from all sides of the public health system that this is a mental illness. For me, this illness is physical but of course it affects me mentally, I am after all a whole human being (MO-7308).”

“They [specialists] don’t take the tests I want, they don’t believe me, they look for other things that are wrong, they overlook the problem, refuse to see the whole person, focus only on their specialism (MO-7619).”

While reflecting on “why”, participants acknowledge the inherent uncertainty in medical knowledge:

“It is all too easy to blame psychological factors. It is often easy to do that when there is a lack of medical knowledge and explanatory models (MO-7202).”

Doctors who falsely reduce their fatigue to a mental condition (as participants see it) put additional strain and burden on patients:

“Health personnel in general all too easily utter the sentence: ‘It is probably something mental’ when there is something they can’t explain. This adds greatly to the burden (MO-7471).”

“I am frustrated by the attitudes people have to the illness, particularly that healthcare personnel are allowed to treat people so badly because they don’t ‘believe in the illness’ and worse, that they put pressure on patients to ‘pull themselves together’ or to undergo treatment that only makes the patient worse. […] To be treated so badly, and also disbelieved and told that one is lazy, has an eating disorder, that it is a matter of willpower etc. when one is seriously ill, is a terrible additional strain (SE-7066).”

Participants interpret psychological explanations as blaming the victim for their ailment, and holding patients responsible for sorting it out:

“In the beginning I was told that I had to push myself, and not to be lazy (MO-7819).”

“In the end I collapsed from going for walks and was told that ‘since I didn’t want to go for walks and take tablets, I could just go home. It was my own fault that I was ill!’ (MO-7210).”

Apart from the frustration of being distrusted, dismissed and blamed, they also describe how the treatment implications of psychological explanations—mainly CBT and physical exercise—are potentially detrimental to their health, as they can cause a (often significant) worsening of their symptoms:

“The GP I had up until that point did not take my illness seriously, and treated me like an unmotivated psychiatric patient […] And therefore gave advice that made me more ill (to increase my activity level and not to rest so much) (MO-7714).”

“CBT can of course generally be used to tackle daily life with the illness, but to offer this as a cure is irresponsible and for me it has resulted in significant worsening (MO-7427).”

“The effect of this [physical exercise] was that in one fell swoop I was on 100% sick leave and very ill for a long time (MO-7819).”

### Theme 5: being dismissed

Participants regularly describe how doctors (especially GPs) try to make themselves unavailable or distance themselves from patients:

“My experience is that they are not available, not interested […] their main concern is to get rid of you (MO-7625).”

“I am treated like a leper (SE-7472).”

“I experienced being treated very badly by the hospital doctor who, after the examination, just laughed at me and said he couldn’t be bothered to waste time on people like me (MO-7803).”

More frequently, our participants recount instances in which doctors (predominantly GPs) had specifically told them to switch doctors, or not to return to them for help:

“Then my GP asked me to switch doctors (MO-7226).”

“In the end she asked me to switch doctors (MI-7772).”

“‘I don’t think they’ll find anything,’ he said. ‘You don’t need to come here anymore’ (MO-7202).”

“She [GP] asked me not to come back to her with this illness. I was not referred—‘no one wants patients like you’ (MI-7337).”

“Completely rejected by the first GP […] He told me off and asked me to find another GP. He was to be pitied himself, working long days, and had such a pain in his own arm. I was not to be pitied, I wasn’t even working, and moreover I could use the private health service if I was in that much pain (MI-7301).”

They also reflect on why doctors’ avoidance behaviours occur:

“Today they are completely helpless when they encounter us, and therefore they react by distancing themselves, both from us as patients and from our illness (MO-7057).”

“ME is generally viewed as a pariah syndrome (MO-7830).”

“… many of them show disdain for patients when they themselves are uncertain and find themselves in unknown territory. Why can’t doctors simply admit that they know little about this—instead of humiliating and mocking the patient when they are uncertain […] that is the way far too many of us are received (MO-7479).”

### Theme 6: fighting the system

Participants explain how doctors (especially GPs) often are unwilling to investigate their symptoms, or refer them to a specialist—sometimes attributing this to doctor beliefs about the psychological understanding of the condition, and the uselessness of further physical examination:

“I found that my GP was so determined that the illness was mentally conditioned that she didn’t feel she needed to investigate any further (MI-7460).”

“The neurological examination he made was superficial, and he asked me nothing about my symptoms, or explored these in any more detail (MO-7670).”

“I myself had to contact the neurologist and specialist […] My GP refused to assess me, or refer me to [hospital name], as they also refused to assess for ME (MO-7462).”

Doctor behaviours and beliefs seem to create a situation in which participants experience being caught in a battle:

“It’s incredible how much I’ve had to fight to be believed, as well as to get what I´m entitled to […] It feels as if I’m fighting an uphill battle, being disbelieved about every single thing (MO-7242).”

“… a battle, to be given the assessment I need and am entitled to (MO-7670).”

“It has been a long battle to be believed and taken seriously (MO-7399).”

“The first 5 years of my disease history were a life of hell, to put it mildly. Weekly battles with the doctor, NAV [Norwegian Labour and Welfare Administration], I was so ill, no help, no guidance, no follow-up (MO-7459).”

Part of the battle relates to perceived punishments (usually lack of care) if patients do not agree with psychological understandings of their ailment:

“… they think it is mental and that we are being quarrelsome if we don’t admit that and get well with cognitive therapy […] In my opinion, follow-up in [name of region] is non-existent if you don’t concur that it is mental (MI-7625).”

Some participants describe a total lack of support:

“… in my case I have received no offers of treatment from the public healthcare service, nor have I been taken seriously (MO-7046).”

“… but there is no help to be had (MI-7375).”

Many participants explain how their experiences in the public health system has made them fearful of doctors or health services altogether. Because of inherent difficulties they face in the national healthcare system, participants able and willing to do so turn to private doctors to receive the help they need:

“I have become fearful of the health service because they totally failed me for many years (MO-7255).”

“I have largely avoided using the public health services, because there is a frightening amount of incompetence (MO-7473).”

“I have used the private health service for the last 3 years, as my public health service GP has never taken my symptoms seriously (MO-7393).”

## Discussion

Despite being written by people of all ages from all over the country, the similarities of participants’ stories are striking. Most importantly, they describe how uncertainties in medical encounters are handled not in collaborative partnership, but in a state of constant battle over the power to define their (the patient’s) situation, as well as measures taken to seek improvements.

### Fighting for their right to self-determination

So far, there has been a lack of attention afforded to the power dimension of service delivery fused by medical uncertainty, and knowledge of how doctors and patients manage uncertainty is fragmented (Atkins, Brownell, Kornelsen, Woollard, & Whiteley, 2013; Han et al., 2011). Such knowledge is important because delivering health services to patients with illness fraught with uncertainty involves heightened potential for the misuse of power, especially on the part of the (usually) most influential actors: doctors (Freidson, 1970; Katz, 1984). The two roles—doctor and patient—constitute an asymmetric power relation. By virtue of their position and professional knowledge, doctors are granted decision-making authority over patients (Freidson, 1970). Medical practice is knowledge applied, and the lack of biomedical knowledge reduces the legitimacy of doctors’ power. This situation calls for collaborative efforts in a partnership of equals, rather than a traditional paternalistic relationship (Lidén, Björk-Brämberg, & Svensson, 2015).

Experiences expressed in our data indicate that doctors handle clinical uncertainties originating from a lack of biomedical knowledge (no causal understanding) and technology (absence of identifiable biomarkers) in a paternalistic manner. The overarching narrative in our data portrays a “fight” and “a long battle” to be listened to, believed and taken seriously. These war metaphors are emblematic of how our participants experience clinical interactions.

The battle appears at three different but mutually dependent levels. At a *micro* level, participants describe being trapped in destructive interactions (“weekly battles with the doctor”) characterized by the “poor”, “awful”, and “dreadful” behaviours (“ridicule”) they encounter. Research on doctors’ attitudes supports these experiences: doctors express suspicion, mistrust and negative stereotyping of these patients (Anderson, Jason, Hlavaty, Porter, & Cudia, 2012; Åsbring & Närvänen, 2003; Donalek, 2009; Raine, Carter, Sensky, & Black, 2004), characterize them as unmotivated and pessimistic (Guise, McVittie, & McKinlay, 2010), and sometimes label them as hypochondriacs (Schoofs, Bambini, Ronning, Bielak, & Woehl, 2004). At a *meso* level, participants depict being trapped in a system that cannot or will not help them (“treatment … is so poor”), a “holding pattern” against which they are “fighting an uphill battle”. At a *macro* level, they depict struggling against perceived “prejudices” and “negative attitudes” in medical culture: typically, the pervasive psychosocial health beliefs about the condition—and of people with ME—prevalent there. Some participants also describe a fight for legitimacy and respect that extends to the wider sociopolitical sphere (“attitudes people have to the illness”).

For the participants, the most contentious issues running through all three levels centre on being met with disbelief and disrespect, inappropriate psychological explanations (combined with limited physical examination) and marginalisation of patient experiences and perspectives. This leads to an experience of being held accountable for their illness and being pressurized to “pull themselves together”, “push myself”, “think positive”, as if it is a “matter of willpower”. If they refuse to comply with treatments, do not get better, or their condition deteriorates, they perceive it is they—not the treatment or clinician—who are held responsible (“told … it was my own fault that I was ill!”).

Our data express personal views, but they also convey the sociocultural context in which participants’ individual experiences are nurtured. By situating their stories in a cultural context we see how they reflect contemporary cultural norms in our society: tiredness is defined as a fault that ought to be handled the “masculine” way, which means: pull yourself together and “act like a man” (Widerberg, 2005), and somatic and mental conditions are hierarchically structured with the latter perceived as both “less real” and as signs of weakness—and therefore almost self-inflicted (Jutel, 2011). Seen in relation this sociocultural context, the main purpose of our participants’ fight appears to be *to preserve their right to self-determination* in relation to defining their situation and preserving their health. Their fight is also about receiving adequate examination and treatment, but that part of their fight is—somewhat surprisingly—far less pronounced.

In line with our findings, people with MUPS conditions often experience being rejected, belittled, ignored, marginalized and stigmatized (Anderson et al., 2012; Bulow, 2008; Donalek, 2009; Gilje, Söderlund, & Malterud, 2008; Kornelsen et al., 2016; Robson & Lian, 2016, 2017; Rosendal et al., 2013; Schoofs et al., 2004; Thomas & Smith, 2005; Widerberg, 2005). Attribution of symptom worsening due to healthcare interactions, however, has not previously been observed, but experiences of detrimental effects of physical therapies and overexertion have (Brown, Khorana, & Jason, 2011; Jason, Benton, Torres-Harding, & Muldowney, 2009). The use of war metaphors has also previously been reported (Davis & Nichter, 2015; Werner, Isaksen, & Malterud, 2004). Contested illnesses can be characterized as ‘‘illnesses you have to fight to get” (Dumit, 2006). Relating the results of our study to previous research indicates that the experiences of our participants are not unique, but quite common among patients with ailments that are medically unexplained and unsubstantiated by biomedical markers.

### Disparate health beliefs

A core element in the battle for self-determination seems to be a lack common conceptual ground in the territory of medical uncertainty, and disparate health beliefs of doctors and patients. Our participants describe how doctors (wrongly) tend to perceive their symptoms as mainly psychological (“they interpret it as mental”, “won’t believe that a person is physically ill”). They explain how doctors (especially GPs) use theories of “mental” aetiology to dismiss or diminish their physical symptoms; and that doctors have told them that they (or people with ME more generally) are “stressed”, “unhappy”, “depressed”, “burnt-out” or “crazy”. This does not accord with the experiences of participants, who describe the condition as being “fairly concrete in physical terms”. Their experiential perspectives are marginalized and disregarded by doctors, who have “a lack of”, “little”, “no”, or “zero understanding” of their ailment, and who dismiss patients’ knowledge of bodily abilities and limitations (“they didn’t believe my situation”). Patients’ experience being met with “disbelief” (“refused to believe that I was ill”), not taken “seriously” (ignored or “brushed aside”), not investigated appropriately (“superficial … he asked me nothing about my symptoms” and “my GP refused to assess me, or refer me”), and accused of “faking” the condition or being a “hypochondriac”. They also regret the energy they waste on “substantiating” their health problems. Underneath these experiences, we find contradictory views of doctor-patient relationships. Each party brings to their encounter a different set of expectations of each other, and patients´ expectations of being invited to a partnership of equals are rarely met.

The strain of disparate health beliefs on doctor-ME patient relationships has previously been described (Bayliss et al., 2014). When doctors attribute MUPS conditions to mental and social causes, patients often perceive it as psychologising and trivialising their symptoms (Anderson et al., 2012; Åsbring & Närvänen, 2004; Rosendal et al., 2013; Schoofs et al., 2004). The lack of biomedical knowledge and efficient medical treatment limits doctors’ ability to help these patients, and this might lead them to distance themselves (Åsbring & Närvänen, 2003). Doctors who encounter patients with ambiguous symptoms have also been shown to make less effort to elucidate and validate symptoms than for those with more straightforward conditions (Epstein et al., 2006).

### Breakdown of doctor-patient partnerships

In many ways, the described battle points to a fundamental breakdown of doctor-patient partnerships. This breakdown is expressed via experiences with doctors who are behaving offensively during consultations (“shouted at”, “yelled at”, “told me off”, “physically threw me out”), and doctors who disassociate and disengage. A common thread is that participants feel disregarded and not listened to by doctors (“absolutely not listened to”, “not interested”, “don’t listen to the patient at all”). Similarly, they depict doctors (especially GPs) who avoid and distance themselves from the patient and their illness (“not available”, “treated like a leper”, “a pariah syndrome”), or (more frequently) they describe doctors who had specifically asked them not to “come back”, or to seek help elsewhere (“don’t … come here anymore”, and “find another GP”). The participants find the attitudes, behaviours and health beliefs of doctors, and, to a lesser extent—the fight against the healthcare system itself– to be “absolutely shocking”, “traumatizing”, and culminating in “a life of hell”.

The breakdown of doctor-patient partnerships is traceable in the language used by participants: they tend to use collective pronouns and identify group characteristics pointing to doctor-patient divisions and the polarized perspectives of the parties involved. Typically, they write about “me and my doctor” or “us (patients) and them”. Participants most often recall specific (enacted) examples of poor treatment experiences involving individual doctors, but frequently refer to how they believe their condition (and people who have it) is *medically* and *culturally* perceived. When doing so, participants use words such as “doctors”, “they”, “their”, “medical profession” and “system”, often contrasted with labels such as “ME patients”, “we”, “us” and “ME sufferers”. Several participants used these labels to directly refer to the “war” they perceive to exist between the parties: “I often have a feeling that most doctors are not on the side of ME patients, on the contrary they are an opponent”. Participants talk about private services differently; often describing that since receiving these “everything has fallen into place”, or even “saved my life”.

Our data show that the breakdown of doctor-patient partnership is not primarily rooted in a lack of clinical comprehension of the disease, or medical cures to treat it. It is of course related to this, but it stems from something more fundamental, and arguably more profound: how doctors are able to communicate that they empathize with, understand and trust (believe) in patients’ own experiences, by treating them in a compassionate humanistic way. As one participant put it: “treating patients/people with the dignity we all deserve”. Interpersonal skills and behaviours (for example, being “friendly, welcoming, attentive and helpful”, “understanding”, and showing “belief”) were often more highly valued by participants in our data than doctors’ medical knowledge: “I am actually very satisfied with my GP; but she knows little about the illness.” Their satisfaction with private services also indicates that it is not primarily the lack of knowledge but how doctors handle this, that is the main source of patients’ discontent.

Although partnerships seem to be weak, participants often acknowledge the difficulties and uncertainties clinicians face when handling “an illness that cannot be determined objectively” in an “unknown territory” where “there is a lack of medical knowledge and explanatory models”. They clearly recognize the limits of biomedical knowledge (“I can’t expect my GP to have this knowledge”) and the doctors’ ability to treat or cure them (“they can *only* offer a diagnosis”, “there is no help to be had”). Participants tend to interpret doctors’ avoidance as stemming from their inability to help (“didn’t know what to do”, “couldn’t do anything”, “completely helpless”), and that “they react by distancing themselves”. These almost empathetic statements indicate that, after all, some partnership still exists.

Participants describe the main obstacles for doctor-patient partnerships as doctors who dismiss patients’ experiences; treat them with disrespect; demonstrate a lack of trust and understanding; falsely describe their fatigue as caused by psychological factors; and give health-damaging treatment advice. The bottom line is that doctors do not acknowledge patients’ experiential perspectives. Patients usually react to this situation by either turning to private providers, or avoiding seeking help. In turn, this leads to a loss of experiential knowledge that could be medically relevant and complement the lack of scientific understanding of this illness.

## Strengths and limitations

Survey participants were drawn from a patient organization, and their identity was never revealed to the researchers. This allowed respondents to describe their experiences without fearing negative consequences from healthcare providers. Giving them an open-ended question and allowing them to describe their experiences in writing—with minimal researcher influence, no time-pressure, and no word-limit—granted them full freedom to reflect on whatever they found most important. Using written texts instead of structured oral interviews, which has been the most common method so far, makes our study original, even in an international context. The relatively high number of participants (more than usual in qualitative studies) is also a strength. Our study is therefore a useful supplement to previous research. The main disadvantage of this method is the lack of face-to-face interaction between researchers and participants, and the lack of opportunity to ask for elaboration of utterances that might have benefited from such.

Collecting free-text qualitative data in a patient satisfaction survey enabled us to combine qualitative and quantitative data on the same issues and—most importantly—from the same patients. Participants reported experiencing ME for a number of (median of 12) years, so they are “experienced” patients. However, their experiences may not be representative of non-organized patients. In addition, men are under-represented in our data. Only 11% of participants rated their condition as severe or very severe, so our data are biased toward higher functioning patients (which is to be expected, as those most seriously affected were probably unable to participate).

We found a significant difference in the initial level of GP satisfaction between those who offered free-text comments and those who did not; and the difference between levels of current GP satisfaction nearly attained significance. It appears that those less satisfied with healthcare services were more likely to offer comments than those who were more satisfied. This, together with our aim to identify the main *obstacles* for good doctor-patient partnerships, implies a focus on negative experiences. This does not necessarily mean our participants do not have positive experiences, but they might have thought it (as did we) more important to report the negative ones, if those aspects are to be improved.

## Conclusion

When ailments cannot be medically identified, explained or alleviated, those who bear them often experience poor healthcare treatment. For those whose symptoms become chronic, the fight for medical, social and political legitimacy begins. Our participants describe such struggles. By using war metaphors like “fight” and “battle”, they describe their engagement with public healthcare services as being caught in a battlefield. The battle is a power struggle over one main question: who is to decide? The rivalry relates to a lack of congruence between doctor and patient perspectives. Patients fight against asymmetric power relations and marginalisation of their perspectives, and for their right to self-determination, but generally to no avail. Some continue to fight; others escape. The main purpose of their fight is to be entitled to participate in the definition and handling of their situation, and to be recognized and respected as autonomous persons with their own understanding, expectations, desires, beliefs, thoughts and emotions in such a way that they preserve their human dignity.

Although the battle is fused by medical uncertainties, the main source of patient contention is not primarily the lack of biomedical knowledge, but how the situation is handled in clinical encounters. Most importantly, doctors seem to fail to communicate acknowledgement of patients’ experiential perspectives and needs. In the name of their professional expertise, it is often considered legitimate for doctors to remove from laymen the right of self-determination (Freidson, 1970). The very foundation for this legitimacy is their professional knowledge. When expert jurisdiction is claimed without the knowledge it ought to rely on, and knowledge is replaced with normative judgements, it becomes ethically problematic. This problem is acknowledged in western societies which now strive to change the paternalistic role of doctors and move towards less asymmetric power relations between doctors and patients through the development of patient-centred care (Cassell, 2010). Patient- or person-centred care is a form of clinical practice based on incorporating the experiential perspective of patients and respecting their autonomy. This perspective is particularly important in the territory of medical uncertainty, where doctor-patient partnership is crucial. Decades of debate and research, recently followed by clinical guidelines emphasising patient-centred clinical methods, is not traceable in the expressed experiences of our study participants. More research is needed to understand why. In the meantime, it might be worth reminding ourselves of an often forgotten fact: explaining the medically unexplained is impossible; understanding it from an experiential perspective is *not*.
